# Trends in Harm Perceptions of E-Cigarettes vs Cigarettes Among Adults Who Smoke in England, 2014-2023

**DOI:** 10.1001/jamanetworkopen.2024.0582

**Published:** 2024-02-28

**Authors:** Sarah E. Jackson, Harry Tattan-Birch, Katherine East, Sharon Cox, Lion Shahab, Jamie Brown

**Affiliations:** 1 Department of Behavioural Science and Health, University College London, London, United Kingdom; 2 SPECTRUM Consortium, Edinburgh, United Kingdom; 3 Department of Addictions, Institute of Psychiatry, Psychology and Neuroscience, King’s College London, United Kingdom

## Abstract

**Importance:**

Electronic cigarettes (e-cigarettes) are less harmful to users than combustible cigarettes. However, public health and media reporting have often overstated the potential risks of e-cigarettes, and inaccurate perceptions of the harms of vaping relative to smoking are pervasive.

**Objective:**

To examine time trends in harm perceptions of e-cigarettes compared with combustible cigarettes among adults who smoke.

**Design, Setting, and Participants:**

This nationally representative monthly cross-sectional survey study was conducted from November 2014 to June 2023 in England. Participants were adults who currently smoke.

**Main Outcomes and Measures:**

Participants were asked whether they thought e-cigarettes were less harmful, equally harmful, or more harmful than cigarettes, or did not know, with the proportion responding less harmful (vs all other responses) as the primary outcome. Logistic regression was used to test associations between survey wave and participants’ perceptions of the harms of e-cigarettes.

**Results:**

Data were collected from 28 393 adults who smoke (mean [SD] age, 43.5 [17.3] years; 13 253 [46.7%] women). In November 2014, 44.4% (95% CI, 42.0%-46.8%) thought e-cigarettes were less harmful than cigarettes, 30.3% (95% CI, 28.2%-32.6%) thought e-cigarettes were equally harmful, 10.8% (95% CI, 9.4%-12.3%) thought they were more harmful, and 14.5% (95% CI, 12.9%-16.4%) did not know. However, by June 2023, the proportion who thought e-cigarettes were less harmful had decreased by 40% (prevalence ratio, 0.60; 95% CI, 0.55-0.66), and the proportion who thought e-cigarettes were more harmful had more than doubled (prevalence ratio, 2.16; 95% CI, 1.84-2.54). Changes over time were nonlinear: late 2019 saw a sharp decline in the proportion who thought e-cigarettes were less harmful and increases in the proportions who thought they were equally or more harmful. These changes were short-lived, returning to pre-2019 levels by the end of 2020. However, perceptions worsened again from 2021 up to the end of the study period: the proportion who thought e-cigarettes were more harmful increased to a new high, and the proportion who thought e-cigarettes were less harmful decreased to levels comparable to those in late 2019. As a result, in June 2023, the perception that e-cigarettes were equally as harmful as cigarettes was the most commonly held view among adults who smoke (33.7%; 95% CI, 31.4%-36.1%), with roughly similar proportions perceiving e-cigarettes to be less (26.7%; 95% CI, 24.6%-28.9%) and more (23.3%; 95% CI, 21.1%-25.7%) harmful.

**Conclusions and Relevance:**

This survey study of adults who smoke in England found that harm perceptions of e-cigarettes have worsened substantially over the last decade, such that most adults who smoked in 2023 believed e-cigarettes to be at least as harmful as cigarettes. The timing of the 2 most notable changes in harm perceptions coincided with the e-cigarette, or vaping product, use-associated lung injury outbreak in 2019 and the recent increase in youth vaping in England since 2021.

## Key Points

**Question:**

How have harm perceptions of electronic cigarettes (e-cigarettes) compared with combustible cigarettes changed since 2014 among adults who smoke in England?

**Findings:**

This survey study of 28 393 adults who smoke found that harm perceptions of e-cigarettes have worsened substantially over the last decade, such that in 2023, most (57.0%) believed e-cigarettes to be equally (33.7%) or more (23.3%) harmful than cigarettes. The timing of the 2 most notable changes in harm perceptions coincided with the e-cigarette, or vaping product, use-associated lung injury outbreak in 2019 and the recent increase in youth vaping in England since 2021.

**Meaning:**

These findings provide evidence of substantial misperceptions about the harms of vaping compared with smoking and underscore the need to clearly communicate the risks so that adults who smoke can make informed choices about the nicotine products they use.

## Teaser

This survey study examines time trends in harm perceptions of electronic cigarettes compared with combustible cigarettes among adults who smoke in England, from 2014 to 2023.

## Introduction

Electronic cigarettes (e-cigarettes) are effective for helping people to stop smoking^1^ and are less harmful than combustible cigarettes (referred to hereafter as cigarettes).^2^ However, many adults who smoke in England (as in many other countries) believe that e-cigarettes are at least as harmful to health as cigarettes,^2,3,4^ which can dissuade adults who smoke from switching to e-cigarettes (and thus reducing their exposure to harmful toxicants).^2^ Several factors may have contributed to increased confusion about the harms of e-cigarettes relative to cigarettes over recent years (see the eAppendix in Supplement 1 for a full literature review). Media reporting often has overstated the risks of e-cigarettes,^5,6^ and evidence suggests that this could exacerbate misperceptions.^2,7^ There has been comparatively little reporting on the deaths caused by smoking; for example, smoking kills approximately 75 000 people each year in England.^8^ Risk messages such as those provided by public health organizations can also change harm perceptions of e-cigarettes.^2,7^ For example, in 2019, there was an outbreak of acute lung injuries that were primarily attributable to vaping contaminated tetrahydrocannabinol products; yet, before the cause was identified, the Centers for Disease Control and Prevention misattributed this to vaping generally and labeled the disease e-cigarette, or vaping product, use-associated lung injury (EVALI).^9^ The following year, COVID-19, a disease that primarily impairs the respiratory system, prompted concerns that e-cigarette use may increase infection risk and disease severity^10,11,12^; however, there is little evidence this was true.^11,13^ Several studies documented short-term increases in the harm perceptions of e-cigarettes following the EVALI outbreak.^2^ How harm perceptions of e-cigarettes have continued to change beyond 2020, in the context of the COVID-19 pandemic (since March 2020) and the growing concern about youth vaping (use of disposable e-cigarettes among young people in England has grown rapidly since June 2021,^14^ leading to calls for a ban on disposable vapes^15^ and widespread news coverage stating potential risks to youth), and the extent to which changes differ between key population subgroups, is not known.

It is important to understand whether there have been changes over time in perceptions of the relative harms of different nicotine products among adults who smoke, because this will have implications for accurate messaging and support. Identifying groups with particularly poor or worsening harm perceptions can inform targeted campaigns to address misperceptions, particularly because campaigns are perceived to negatively portray vaping.^16^ Using data from a nationally representative survey in England, this study aimed to examine how harm perceptions of e-cigarettes compared with cigarettes have changed over time among adults who currently smoke, and the extent to which changes have differed by age, socioeconomic position, and vaping status (variables known to be associated with smoking, vaping, and harm perceptions of e-cigarettes vs cigarettes).^17,18,19,20^

## Methods

The study protocol and analysis plan for this survey study were preregistered on Open Science Framework^21^ and were followed without amendment. Ethical approval for the Smoking and Alcohol Toolkit Study was granted originally by the University College London ethics committee. The data are collected by Ipsos Mori and are anonymized when received by University College London. All participants provided informed verbal consent. The study conformed to the American Association for Public Opinion Research (AAPOR) reporting guidelines for survey research.

### Design

Data were drawn from the ongoing Smoking Toolkit Study, a monthly cross-sectional survey of a representative sample of adults in England.^22^ The study uses a hybrid of random probability and simple quota sampling to select a new sample of approximately 1700 adults each month. Comparisons with other national surveys and sales data indicate that sociodemographic characteristics, smoking prevalence, and cigarette consumption are nationally representative.^22,23^

Data were initially collected through face-to-face computer-assisted interviews. However, social distancing restrictions under the COVID-19 pandemic meant that no data were collected in March 2020, and data from April 2020 onward were collected via telephone. The telephone-based data collection uses sampling and weighting approaches similar to the face-to-face interviews, and comparisons of the 2 data collection modalities indicate good comparability.^24,25,26^

For the present study, we used data from survey respondents between November 2014 (the first wave to assess harm perceptions of e-cigarettes) and June 2023 (the most recent data available at the time of analysis). We restricted our sample to those aged 18 years or older who reported current smoking (see the Measures section), because the item assessing harm perceptions of e-cigarettes was only asked to those who smoke.

### Measures

Smoking status was assessed with the question, “Which of the following best applies to you? Please note we are referring to cigarettes and other kinds of tobacco that you set light to and NOT electronic or ‘heat-not-burn’ cigarettes?: (a) I smoke cigarettes (including hand-rolled) every day; (b) I smoke cigarettes (including hand-rolled), but not every day; (c) I do not smoke cigarettes at all, but I do smoke tobacco of some kind (eg, pipe, cigar, or shisha); (d) I have stopped smoking completely in the last year; (e) I stopped smoking completely more than a year ago; (f) I have never been a smoker (ie, smoked for a year or more).” Responses a, b, and c were considered current smoking. Those who responded d, e, or f were excluded from the sample.

Harm perceptions of e-cigarettes was assessed with the question, “Compared to regular cigarettes, do you think electronic cigarettes are more, less, or equally harmful to health?” Response options were “more harmful,” “less harmful,” “equally harmful,” or “don’t know.” We analyzed the proportion responding less harmful (vs all other responses) as our primary outcome, consistent with current evidence that e-cigarettes are less harmful than cigarettes,^2^ and the proportions responding equally harmful (vs all other), more harmful (vs all other), and don’t know (vs all other) as secondary outcomes. We conducted sensitivity analyses with don’t know responses excluded.

Age was categorized as 18 to 34 years, 35 to 64 years, and 65 years or older. Occupational social grade was categorized as ABC1 (managerial, professional, or intermediate) and C2DE (skilled manual workers, semiskilled and unskilled manual workers, state pensioners, casual and lowest grade workers, and unemployed with state benefits only).

Vaping status was assessed with a series of questions that ask participants whether they are using an e-cigarette or vaping device to help them stop smoking, cut down the amount smoked, in situations when smoking is not permitted, or for any other reason at all. Those who reported e-cigarette use in response to any of these questions were considered current vapers.

### Statistical Analysis

Data were analyzed using R statistical software version 4.2.2 (R Project for Statistical Computing). We excluded participants with missing data on harm perceptions.

The Smoking Toolkit Study uses raking to weight the sample to match the population in England on age, social grade, region, housing tenure, ethnicity, and working status within sex. This profile is determined monthly by combining data from the UK Census, the Office for National Statistics midyear estimates, and the annual National Readership Survey.^22^ The following analyses used weighted data.

We used logistic regression to test associations between survey wave and perception of e-cigarettes as (a) less harmful than cigarettes (primary outcome), and (b) equally harmful, (c) more harmful, and (d) don’t know (secondary outcomes). Survey wave was modeled using restricted cubic splines with 5 knots, to allow relationships with time to be flexible and nonlinear.

To explore moderation by age, occupational social grade, and vaping status, we repeated the models including the interaction between the moderator of interest and survey wave, thus allowing time trends to differ across subgroups. Each interaction was tested in a separate model. Two-sided *P *< .05 was considered statistically significant.

We used estimates from our models to plot the estimated prevalence of each harm perception over the study period (overall and by moderating variables), alongside unmodeled observed (weighted) data aggregated by quarter (to increase sample size contributing to each data point and reduce noise). We also used our modeled estimates to derive prevalence ratios (PRs) for the change in prevalence across the whole time series (June 2023 vs November 2014) alongside 95% CIs calculated using bootstrapping.

## Results

Of 169 433 participants surveyed in eligible waves, 28 393 (16.8%) reported current smoking. There were no missing data on e-cigarette harm perceptions (or age, occupational social grade, or vaping status), leaving a final sample for analysis of 28 393 adults who smoke (mean [SD] age, 43.5 [17.3] years; 13 253 [46.7%] women; 15 415 [54.3%] social grades C2DE; 5879 [20.7%] reported current vaping).

### Overall Estimates of Harm Perceptions

Table 1 shows descriptive data on harm perceptions, aggregated across survey waves (corresponding estimates excluding those who responded don’t know from the sample are shown in eTable 1 in Supplement 1). Overall, 35.2% (95% CI, 34.6%-35.8%) of adults who smoke perceived e-cigarettes to be less harmful than cigarettes, 36.7% (95% CI, 36.0%-37.3%) said it was equally harmful, 13.4% (95% CI, 12.9%-13.8%) said it was more harmful, and 14.8% (95% CI, 14.3%-15.2%) did not know. The proportion who thought e-cigarettes were less harmful was higher among those who currently vaped (56.7% [95% CI, 55.4%-58.1%] vs 29.4% [95% CI, 28.8%-30.1%] among those who did not currently vape), those from more advantaged social grades ABC1 (41.9% [95% CI, 41.0%-42.9%] vs 30.7% [95% CI, 29.9%-31.5%] among social grades C2DE), and those aged 35 to 64 years (37.6% [95% CI, 36.7%-38.5%] vs 34.1% [95% CI, 33.1%-35.0%] among those aged 18-34 years and 29.2% [95% CI, 95% CI, 27.7%-30.7%] among those aged ≥65 years). Younger participants (18-34 years) were more likely than middle-aged (35-64 years) and older (≥65 years) participants to perceive e-cigarettes as equally or more harmful than cigarettes, whereas older participants (≥65 years) were more likely to say they did not know. Approximately one-third (34.9% [95% CI, 33.6%-36.2%]) of dual users (who both smoked and vaped) reported perceiving that e-cigarettes were equally or more harmful than cigarettes.

**Table 1.** Harm Perceptions, Aggregated Across Survey Waves^a^

Variable	Respondents, % (95% CI) (unweighted N = 28 393)			
	Less harmful	Equally harmful	More harmful	Don’t know
All adults who smoke	35.2 (34.6-35.8)	36.7 (36.0-37.3)	13.4 (12.9-13.8)	14.8 (14.3-15.2)
Age group, y				
18-34	34.1 (33.1-35.0)	40.0 (39.0-41.0)	16.0 (15.2-16.7)	10.0 (9.4-10.6)
35-64	37.6 (36.7-38.5)	34.8 (33.9-35.6)	12.0 (11.4-12.6)	15.6 (15.0-16.3)
≥65	29.2 (27.7-30.7)	32.8 (31.3-34.4)	9.8 (8.9-10.8)	28.1 (26.6-29.7)
Occupational social grade^b^				
ABC1 (more advantaged)	41.9 (41.0-42.9)	33.3 (32.5-34.2)	10.6 (10.1-11.2)	14.1 (13.5-14.7)
C2DE (less advantaged)	30.7 (29.9-31.5)	38.9 (38.0-39.7)	15.2 (14.6-15.8)	15.2 (14.6-15.8)
Vaping status				
Nonvaping	29.4 (28.8-30.1)	38.8 (38.1-39.5)	15.3 (14.8-15.8)	16.5 (16.0-17.0)
Current vaping	56.7 (55.4-58.1)	28.7 (27.4-29.9)	6.2 (5.6-6.9)	8.4 (7.6-9.1)

^a^ Corresponding estimates excluding those who responded don’t know from the sample are shown in eTable 1 in Supplement 1.

^b^ ABC1 refers to managerial, professional, or intermediate workers. C2DE refers to skilled manual workers, semiskilled and unskilled manual workers, state pensioners, casual and lowest grade workers, and those who are unemployed with state benefits only.

### Changes in Harm Perceptions From the Start to the End of the Study Period

**Figure 1.**
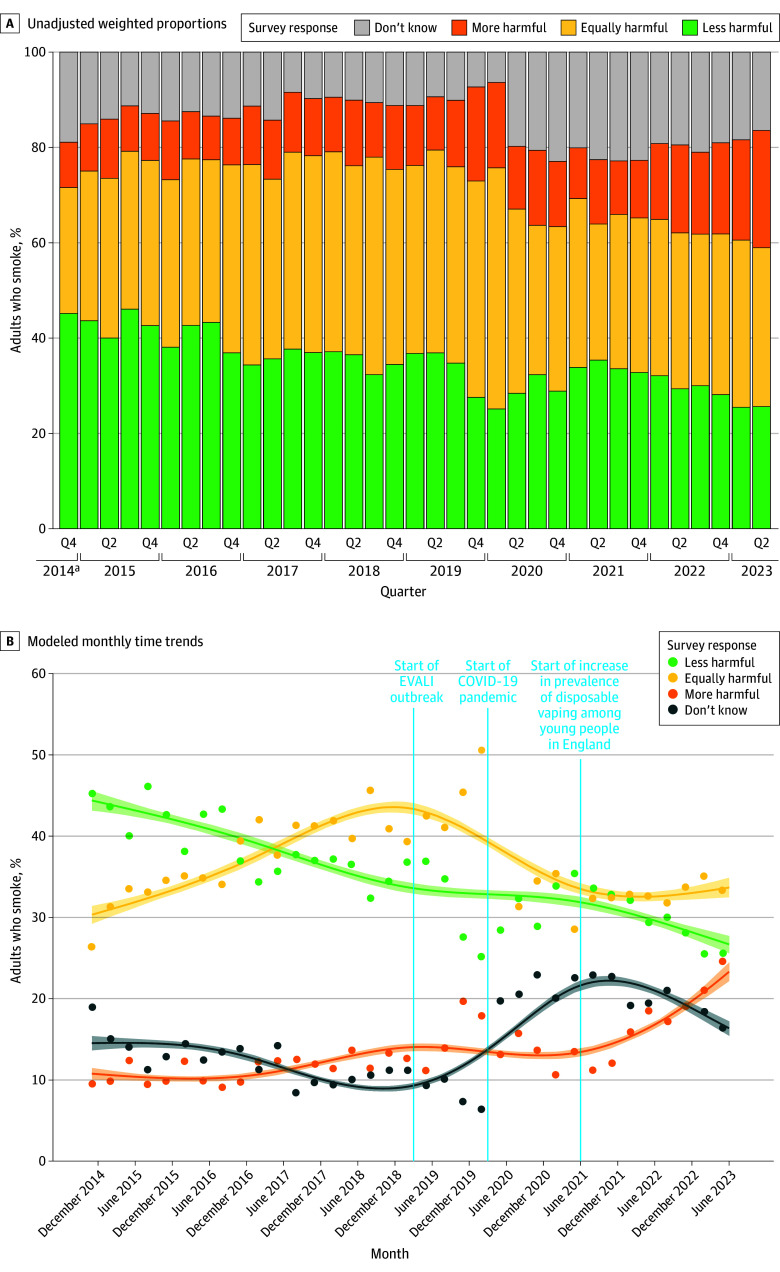
Harm Perceptions of E-Cigarettes vs Cigarettes Among 28 393 Adults Who Smoke in England, November 2014 to June 2023 Panel A shows unadjusted weighted proportions aggregated by quarter (Q). Panel B shows modeled monthly time trends: lines represent modeled weighted prevalence by monthly survey wave (modeled nonlinearly using restricted cubic splines, 5 knots), shaded bands represent SEs, and points represent observed weighted prevalence by quarter. Vertical lines indicate the timing of the start of the e-cigarette, or vaping product, use-associated lung injury (EVALI) outbreak (March 2019),^9^ COVID-19 pandemic (March 2020), and the rapid increase in prevalence of disposable vaping among young people in England (June 2021).^14^ Corresponding figures excluding don’t know responses are provided in eFigure 1 in Supplement 1. ^a^Quarter 4 2014 includes data from November to December only.

Figure 1A shows descriptive data on e-cigarette harm perceptions across the study period, aggregated by quarter (corresponding figures excluding don’t know responses are provided in eFigure 1 in Supplement 1). Models revealed significant changes in harm perceptions between the start and end of the study period (Table 2 and Table 3) (corresponding estimates excluding those who responded don’t know from the sample are shown in eTable 2 in Supplement 1). In November 2014, when e-cigarette harm perceptions were first assessed, the most common perception among adults who smoke was that e-cigarettes were less harmful than cigarettes (44.4%; 95% CI, 42.0%-46.8%), similar to the proportions perceiving e-cigarettes to be equally harmful (30.3%; 95% CI, 28.2%-32.6%) or more harmful (10.8%; 95% CI, 9.4%-12.3%) combined, with 14.5% (95% CI, 12.9%-16.4%) saying they did not know. However, by June 2023, the proportion who thought e-cigarettes were less harmful had decreased by 40% (PR, 0.60; 95% CI, 0.55-0.66) and the proportion who thought they were more harmful had more than doubled (PR, 2.16; 95% CI, 1.84-2.54). Hence, in June 2023, the perception that e-cigarettes were equally as harmful as cigarettes was the most commonly held view among adults who smoke (33.7%; 95% CI, 31.4%-36.1%), with roughly similar proportions perceiving e-cigarettes to be less (26.7%; 95% CI, 24.6%-28.9%) and more (23.3%; 95% CI, 21.1%-25.7%) harmful.

**Table 2.** Changes in Harm Perceptions (Less Harmful and Equally Harmful) From the Start to the End of the Study

Variable	Less harmful			Equally harmful		
	Respondents, % (95% CI)^a^		PR (95% CI)^b^	Respondents, % (95% CI)^a^		PR (95% CI)^b^
	November 2014	June 2023		November 2014	June 2023	
All adults who smoke	44.4 (42.0-46.8)	26.7 (24.6-28.9)	0.60 (0.55-0.66)	30.3 (28.2-32.6)	33.7 (31.4-36.1)	1.11 (1.01-1.22)
Age group, y						
18-34	41.0 (37.3-44.8)	26.5 (23.1-30.1)	0.65 (0.55-0.75)	34.9 (31.3-38.5)	35.2 (31.4-39.2)	1.01 (0.87-1.16)
35-64	49.4 (45.9-52.9)	28.1 (25.1-31.3)	0.57 (0.50-0.65)	26.6 (23.7-29.7)	33.1 (29.8-36.5)	1.24 (1.08-1.43)
≥65	32.8 (26.7-39.4)	21.8 (17.1-27.4)	0.67 (0.49-0.91)	31.4 (25.7-37.7)	31.6 (25.5-38.5)	1.01 (0.76-1.33)
Occupational social grade^c^						
ABC1 (more advantaged)	53.4 (49.3-57.4)	28.0 (25.3-30.9)	0.53 (0.47-0.59)	24.7 (21.6-28.2)	34.4 (31.4-37.6)	1.39 (1.19-1.62)
C2DE (less advantaged)	39.8 (36.8-42.8)	25.6 (22.6-28.9)	0.64 (0.56-0.74)	33.3 (30.6-36.2)	33.2 (29.8-36.7)	1.00 (0.87-1.13)
Vaping status						
Nonvaping	39.4 (36.8-42.1)	19.0 (17.0-21.3)	0.48 (0.43-0.55)	32.4 (30.0-34.9)	33.4 (30.7-36.3)	1.03 (0.93-1.15)
Current vaping	63.2 (57.8-68.4)	41.6 (37.1-46.2)	0.66 (0.57-0.75)	22.4 (18.2-27.3)	35.0 (30.6-39.5)	1.56 (1.24-1.97)

Abbreviation: PR, prevalence ratio.

^a^ Weighted prevalence was calculated from logistic regression models on all adults who smoke and (for estimates by age, occupational social grade, and vaping status) allowing an interaction between survey wave and the moderator of interest, modeled nonlinearly using restricted cubic splines. Corresponding estimates excluding those who responded don’t know from the sample are shown in eTable 2 in Supplement 1.

^b^ PR refers to the change in responses between November 2014 and June 2023.

^c^ ABC1 refers to managerial, professional, or intermediate workers. C2DE refers to skilled manual workers, semiskilled and unskilled manual workers, state pensioners, casual and lowest grade workers, and those who are unemployed with state benefits only.

**Table 3.** Changes in Harm Perceptions (More Harmful and Don’t Know) From the Start to the End of the Study

Variable	More harmful			Don’t know		
	Respondents, % (95% CI)^a^		PR (95% CI)^b^	Respondents, % (95% CI)^a^		PR (95% CI)^b^
	November 2014	June 2023		November 2014	June 2023	
All adults who smoke	10.8 (9.4-12.3)	23.3 (21.1-25.7)	2.16 (1.84-2.54)	14.5 (12.9-16.4)	16.4 (14.7-18.2)	1.13 (0.96-1.32)
Age group, y						
18-34	14.9 (12.4-17.7)	29.3 (25.4-33.5)	1.97 (1.60-2.43)	9.3 (7.2-11.9)	9.8 (7.7-12.5)	1.05 (0.76-1.46)
35-64	8.8 (7.1-10.9)	21.4 (18.4-24.8)	2.43 (1.90-3.16)	15.2 (12.8-17.9)	17.4 (15.0-20.1)	1.15 (0.92-1.42)
≥65	4.5 (2.5-8.1)	11.6 (8.2-16.2)	2.56 (1.41-5.27)	31.9 (25.6-38.9)	34.0 (28.0-40.5)	1.07 (0.82-1.42)
Occupational social grade^c^						
ABC1 (more advantaged)	6.8 (5.2-8.9)	21.7 (18.9-24.7)	3.17 (2.39-4.40)	15.2 (12.3-18.6)	16.4 (14.2-18.8)	1.08 (0.86-1.39)
C2DE (less advantaged)	12.9 (11.0-15.0)	24.7 (21.5-28.3)	1.92 (1.58-2.35)	14.1 (12.2-16.4)	16.4 (14.0-19.1)	1.16 (0.95-1.44)
Vaping status						
Nonvaping	12.0 (10.4-13.8)	27.7 (24.9-30.6)	2.30 (1.96-2.73)	16.2 (14.3-18.4)	19.9 (17.7-22.3)	1.23 (1.04-1.44)
Current vaping	6.2 (3.9-9.6)	15.3 (11.8-19.5)	2.47 (1.54-4.21)	8.2 (5.5-12.1)	9.2 (6.9-12.2)	1.13 (0.72-1.89)

Abbreviation: PR, prevalence ratio.

^a^ Weighted prevalence was calculated from logistic regression models on all adults who smoke and (for estimates by age, occupational social grade, and vaping status) allowing an interaction between survey wave and the moderator of interest, modeled nonlinearly using restricted cubic splines. Corresponding estimates excluding those who responded don’t know from the sample are shown in eTable 2 in Supplement 1.

^b^ PR refers to the change in responses between November 2014 and June 2023.

^c^ ABC1 refers to managerial, professional, or intermediate workers. C2DE refers to skilled manual workers, semiskilled and unskilled manual workers, state pensioners, casual and lowest grade workers, and those who are unemployed with state benefits only.

### Time Trends in Harm Perceptions

Changes in harm perceptions over time were nonlinear (Figure 1B). From November 2014 to July 2019, the proportion of adults who smoke who thought e-cigarettes were less harmful than cigarettes declined steadily to 33.2% (95% CI, 32.0%-34.5%) and the proportion who thought they were equally harmful increased to 42.5% (95% CI, 41.1%-43.8%). The proportions who thought they were more harmful or who did not know were relatively stable up to the end of 2016; the former increased over subsequent years to a high of 14.1% (95% CI, 13.1%-15.1%) in April 2019, and the latter decreased to a low of 8.9% (95% CI, 8.3%-9.7%) in October 2018.

**Figure 2.**
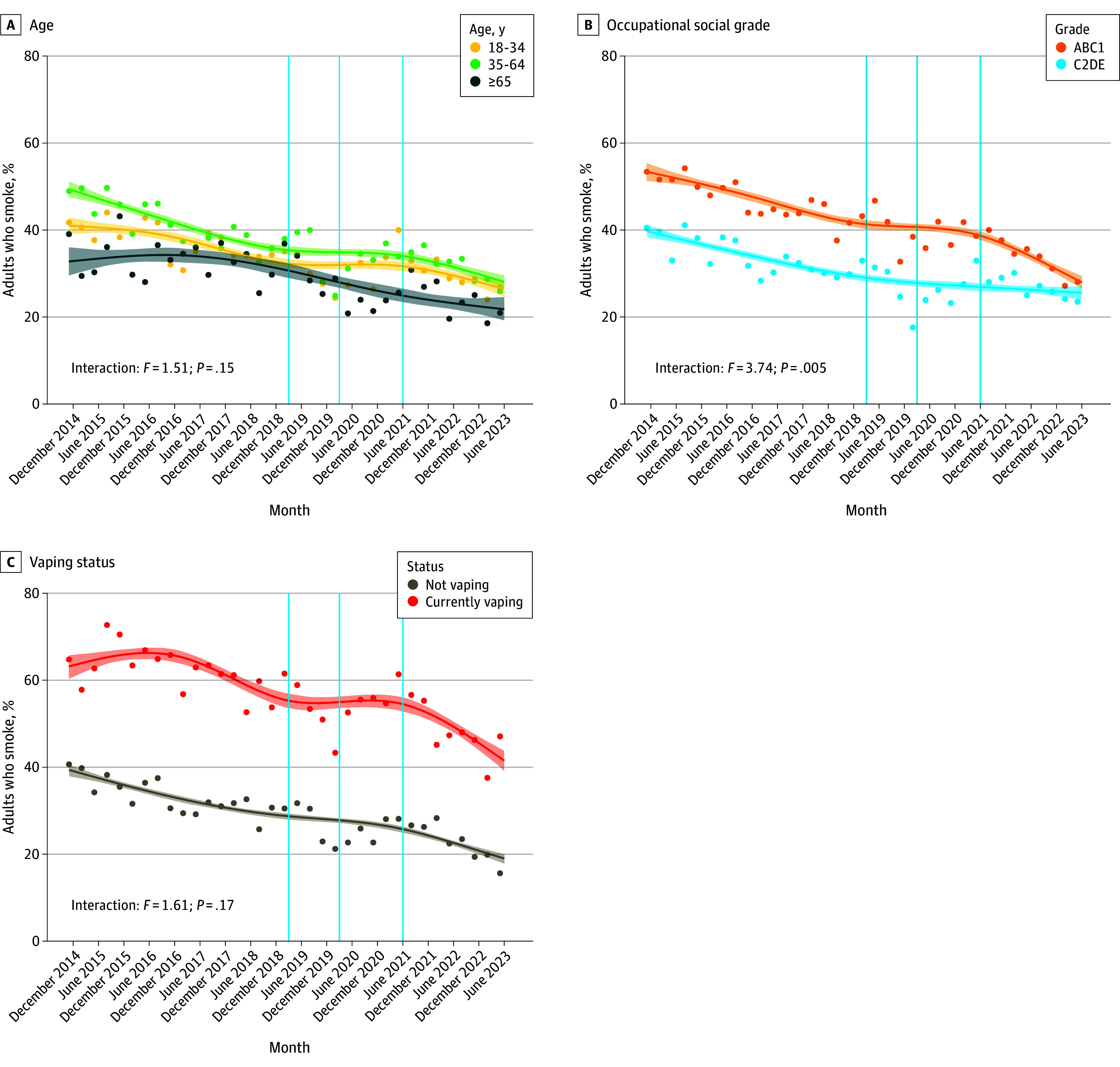
Trends in the Perception of E-Cigarettes as Less Harmful Than Cigarettes Among Adults Who Smoke in England Graphs show data by age (A), occupational social grade (B; ABC1 refers to managerial, professional, or intermediate workers, and C2DE refers to skilled manual workers, semiskilled and unskilled manual workers, state pensioners, casual and lowest grade workers, and those who are unemployed with state benefits only), and vaping status (C). Lines represent point estimates from logistic regression allowing an interaction between age and survey wave, modeled nonlinearly using restricted cubic splines (5 knots). Shaded bands represent SEs. Points represent observed weighted prevalence by quarter. From left to right, vertical lines indicate the timing of the start of the e-cigarette, or vaping product, use-associated lung injury outbreak (March 2019),^9^ COVID-19 pandemic (March 2020), and the rapid increase in prevalence of disposable vaping among young people in England (June 2021).^14^ Figures for trends in the perception of e-cigarettes as equally harmful, more harmful, or don’t know are shown in eFigure 2 in Supplement 1. Corresponding figures excluding those who responded “don’t know” from the sample are shown in eFigure 3 in Supplement 1.

Inspection of the unmodeled data points (ie, observed weighted prevalence by quarter, shown as points in Figure 1 and Figure 2) suggests there was then a marked shift in perceptions in late 2019: a sharp decline in the proportion who thought e-cigarettes were less harmful than cigarettes to a low of 25.1% (95% CI, 21.0%-29.3%) in quarter 1 of 2020 and increases in the proportions who thought they were equally or more harmful, reaching highs of 50.6% (95% CI, 45.7%-55.5%) in quarter 1 of 2020 and 19.7% (95% CI, 16.6%-22.9%) in quarter 4 of 2019 (Figure 1B). Changes in the proportions who thought e-cigarettes were less or more harmful were short-lived, returning to pre-2019 levels by the end of 2020. However, the proportion who thought e-cigarettes were equally harmful decreased to below 2018 levels and remained lower, offset by an increase in the proportion who did not know how the harms of e-cigarettes compared with cigarettes in quarter 2 of 2020 (Figure 1B). From 2021 to the end of the study period in mid-2023, the proportion who thought e-cigarettes were more harmful than cigarettes increased to a new high (surpassing the previous peak in the unmodeled data points in late 2019), the proportion who thought they were less harmful decreased to levels comparable to those in late 2019, the proportion who thought they were equally harmful was stable, and the proportion who did not know declined (Figure 1B).

### Interactions With Age, Occupational Social Grade, and Vaping Status

The decline over the study period in the proportion who thought e-cigarettes were less harmful than cigarettes was similar across age groups (Figure 2A). The decline in the proportion who thought e-cigarettes were equally harmful since 2019 was most pronounced among the oldest group (≥65 years) and least pronounced among the youngest group (18-34 years; *P* for interaction = .001) (eFigure 2A in Supplement 1). The increase in the proportion who thought e-cigarettes were more harmful since 2021 was most pronounced among the youngest group and was absent in the oldest group (*P* for interaction = .001) (eFigure 2B in Supplement 1).

The decline in the proportion who thought e-cigarettes were less harmful than cigarettes since 2021 was more pronounced among social grades ABC1, with less change observed for C2DE (*P* for interaction = .005) (Figure 2B). Over the same period, there was also an increase in the proportion who thought e-cigarettes were equally harmful among social grades ABC1 but not C2DE (*P* for interaction = .003) (eFigure 2D in Supplement 1). By the end of the study period, the inequality gap in harm perceptions had closed, such that those from social grades ABC1 had perceptions similar to those from C2DE.

There were similar declines in the proportion who thought e-cigarettes were less harmful by vaping status across the period (Figure 2C). Notably, the proportion who believed e-cigarettes were less harmful was consistently lower among those who did not vape, and only 19.0% (95% CI, 17.0%-21.3%) of this group thought this by June 2023. There was an increase in the proportion who thought e-cigarettes were equally harmful since 2021 among those who currently vaped but no change among those who smoked but did not vape (eFigure 2G in Supplement 1). As a result, in June 2023, 33.4% (95% CI, 30.7%-36.3%) of participants who smoked but did not vape thought e-cigarettes were equally harmful as cigarettes, 27.7% (95% CI, 24.9%-30.6%) thought they were more harmful, and 19.9% (95% CI, 17.7%-22.3%) were unsure. Corresponding figures excluding those who responded don’t know from the sample are shown in eFigure 3 in Supplement 1.

## Discussion

This survey study found that among adults who smoke in England, harm perceptions of e-cigarettes compared with cigarettes have worsened considerably over the past decade. In 2014, the most common perception was that e-cigarettes were less harmful than cigarettes. However, by June 2023, the proportion who thought e-cigarettes were less harmful had decreased by 40% and the proportion who thought they were more harmful had more than doubled. Although perceptions were generally more positive among those aged 35 to 64 years, those from more advantaged social grades, and those who currently vaped, deterioration was observed across all subgroups. As a result, only a minority (26.7%) of adults who smoke now think e-cigarettes are less harmful than cigarettes, including only 19.0% of smokers who do not vape, who would most benefit from switching to a reduced harm product. More than one-half (57.0%) of all adults surveyed think e-cigarettes are equally (33.7%) or more (23.3%) harmful than cigarettes. Among those who did not vape, 61.1% overall think e-cigarettes are equally (33.4%) or more (27.7%) harmful than cigarettes, and 19.9% are unsure.

Changes over time were nonlinear. Consistent with previous studies,^2,27,28^ including from the same data set up to 2019,^4^ we observed a sharp decline in late 2019 in the proportion who thought e-cigarettes were less harmful than cigarettes and increases in the proportions who thought e-cigarettes were equally or more harmful. This coincided with the timing of the EVALI outbreak, which was at its peak in September 2019.^9^ EVALI cases declined to virtually zero by February 2020, at which point the Centers for Disease Control and Prevention stopped publishing updates on case numbers.^9^ Likewise, changes in the proportions who thought e-cigarettes were less or more harmful were short-lived, returning to pre-2019 levels by the end of 2020.^27^ Interestingly, changes in harm perceptions around the timing of EVALI appeared similar across age groups, despite EVALI predominantly affecting young people^9^ and this being highlighted in media coverage at the time.^5^

Following EVALI, the COVID-19 pandemic did not coincide with substantial changes in harm perceptions of e-cigarettes, with the only notable change being an increase in don’t know responses. This may be linked to an increase in confusion about public health risks resulting from misinformation transmitted during the pandemic.^29^ However, perceptions worsened again from 2021 to the end of the study period. The proportion who thought e-cigarettes were more harmful increased to a new high (surpassing the previous peak in late 2019), and the proportion who thought they were less harmful decreased to levels comparable to those from late 2019. These changes coincided with the timing of concerns about an increase in youth vaping in England^30^ since new disposable e-cigarettes have become popular.^14^ This trend has been widely reported in the media (with a substantial increase in news reporting of vaping in 2022 and 2023)^31^ and is a priority issue for policymakers^32^ and practitioners.^15^ We observed a clear age gradient in changing harm perceptions since 2021, with the increase in the proportion who thought e-cigarettes were more harmful than cigarettes most pronounced among those younger than 35 years and absent in those older than 65 years. This aligns with the emphasis on risks to young people in reports on youth vaping.^15^ It suggests there is a disconnect between young people’s risk perceptions of e-cigarettes and their behavior (ie, that use of e-cigarettes is more common among younger age groups, despite a larger proportion thinking they are at least as harmful as smoking).

The deterioration in evidence-based harm perceptions since 2021 was also much more prominent among those from more advantaged social grades, with little change among less advantaged social grades. Overall, the more advantaged social grades held more positive views of e-cigarettes’ relative harms. However, the decline since 2021 closed the inequality gap, bringing their perceptions in line with the (more negative) views of those from less advantaged social grades. Although reducing inequalities is important, it does not improve public health unless it is achieved by correcting misperceptions in the less advantaged group, rather than worsening in the more advantaged group.

These findings have important implications for public health. Misperceptions about the risks of e-cigarettes compared with cigarettes are a health risk in and of themselves. If people who smoke think vaping is equally or more harmful than smoking, they may be less inclined to try and switch to vaping, leaving them using a more harmful product.^2^ In April 2023, the English government announced a national swap to stop campaign, which aims to offer 1 million people a free vaping starter kit with behavioral support, to help them quit smoking.^33^ Strategies such as these could be undermined if people who smoke are unwilling to try vaping because of safety concerns. In addition, if people who both smoke and vape (dual users) think the risks are similar, they may not see any benefit of stopping smoking and instead continue both behaviors. Our data suggest that 1 in 2 dual users now think e-cigarettes are equally or more harmful than cigarettes, so there is substantial opportunity to correct misperceptions. Similarly, if young vapers who have never smoked think the risks are similar, they may be equally likely to start smoking as to start vaping. To date, smoking prevalence among young people in England has remained low despite an increase in vaping prevalence,^2^ although there are warning signs that the decline in smoking may have leveled off or reversed in recent years.^2,34^ There is a need to clearly communicate the risks of vaping compared with smoking to ensure this pattern does not change.

### Limitations

This study had several limitations. Only adults who currently smoke were asked about harm perceptions of e-cigarettes, so we were unable to explore changes among nonsmokers or youth. We included all adults who reported current smoking but did not separate noncigarette tobacco (eg, cigars and pipes) users, who may hold different harm perceptions compared with those who smoke cigarettes, because of their small sample size. In addition, findings are unlikely to generalize beyond England; cross-national comparisons could help to understand potential factors contributing to changing vaping perceptions. Although we speculated on the potential causes of the changes in harm perceptions we observed, further research (eg, qualitative) is required to provide deeper insight into the factors that have caused people’s perceptions of e-cigarettes to change and why changes have been different across population subgroups. Further research could explore differences in intersectional subgroups (eg, age by socioeconomic position) to gain more nuanced insights into unequal harm perceptions.

## Conclusions

Harm perceptions of e-cigarettes have worsened substantially over the last decade, such that the vast majority of adults who smoke and do not vape in England do not believe e-cigarettes are less harmful than cigarettes. The timing of the most notable changes in harm perceptions coincided with the EVALI outbreak and the recent increase in youth vaping.

## Notes

Supplement 1. **eAppendix. **Literature Review
eReferences
**eTable 1.** Harm Perceptions, Aggregated Across Survey Waves, Excluding Don’t Know Responses (Unweighted n=24,088) **eFigure 1.** Harm Perceptions of e-Cigarettes Compared With Cigarettes Among Adults Who Smoke in England, November 2014 to June 2023, Excluding Don’t Know Responses (n=24,088) **eTable 2.** Changes in Harm Perceptions From the Start to the End of the Study, Excluding Don’t Know Responses **eFigure 2.** Trends in Harm Perceptions of e-Cigarettes Compared With Cigarettes Among Adults Who Smoke in England, by Age (A-C), Occupational Social Grade (D-F), and Vaping Status (G-I), November 2014 to June 2023: Equally Harmful, More Harmful, and Don’t Know **eFigure 3.** Trends in Harm Perceptions of e-Cigarettes Compared With Cigarettes Among Adults Who Smoke in England, by Age (A-C), Occupational Social Grade (D-F), and Vaping Status (G-I), November 2014 to June 2023, Excluding Don’t Know Responses

Supplement 2.
Data Sharing Statement
