# Supplementary material for: Trends in Harm Perceptions of E-Cigarettes vs Cigarettes Among Adults Who Smoke in England, 2014-2023
Source: JAMA Netw Open. 2024 Feb 28;7(2):e240582. doi: 10.1001/jamanetworkopen.2024.0582 (PMC10902732; doi:10.1001/jamanetworkopen.2024.0582)
Supplement: Supplement 1. — eAppendix. Literature Review eReferences eTable 1. Harm Perceptions, Aggregated Across Survey Waves, Excluding Don’t Know Responses (Unweighted n=24,088) eFigure 1. Harm Perceptions of e-Cigarettes Compared With Cigarettes Among Adults Who Smoke in England, November 2014 to June 2023, Excluding Don’t Know Responses (n=24,088) eTable 2. Changes in Harm Perceptions From the Start to the End of the Study, Excluding Don’t Know Responses eFigure 2. Trends in Harm Perceptions of e-Cigarettes Compared With Cigarettes Among Adults Who Smoke in England, by Age (A-C), Occupational Social Grade (D-F), and Vaping Status (G-I), November 2014 to June 2023: Equally Harmful, More Harmful, and Don’t Know eFigure 3. Trends in Harm Perceptions of e-Cigarettes Compared With Cigarettes Among Adults Who Smoke in England, by Age (A-C), Occupational Social Grade (D-F), and Vaping Status (G-I), November 2014 to June 2023, Excluding Don’t Know Responses [file jamanetwopen-e240582-s001.pdf]

Supplemental Online Content

Jackson SE, Tattan-Birch H, East K, Cox S, Shahab L, Brown J. Trends in harm perceptions of e-cigarettes vs cigarettes among adults who smoke in England, 2014-2023. *JAMA Netw Open*. 2024;7(2):e240582. doi:10.1001/jamanetworkopen.2024.0582

eAppendix. Literature Review

eReferences

eTable 1. Harm Perceptions, Aggregated Across Survey Waves, Excluding Don’t Know Responses (Unweighted n=24,088)

eFigure 1. Harm Perceptions of e-Cigarettes Compared With Cigarettes Among Adults Who Smoke in England, November 2014 to June 2023, Excluding Don’t Know Responses (n=24,088)

eTable 2. Changes in Harm Perceptions From the Start to the End of the Study, Excluding Don’t Know Responses

eFigure 2. Trends in Harm Perceptions of e-Cigarettes Compared With Cigarettes Among Adults Who Smoke in England, by Age (A-C), Occupational Social Grade (D-F), and Vaping Status (G-I), November 2014 to June 2023: Equally Harmful, More Harmful, and Don’t Know

eFigure 3. Trends in Harm Perceptions of e-Cigarettes Compared With Cigarettes Among Adults Who Smoke in England, by Age (A-C), Occupational Social Grade (D-F), and Vaping Status (G-I), November 2014 to June 2023, Excluding Don’t Know Responses

This supplemental material has been provided by the authors to give readers additional information about their work.

© 2024 Jackson SE et al. *JAMA Network Open.*

eAppendix. Literature Review

The US EVALI outbreak caused over 2,800 hospitalisations and 68 deaths, mostly among people under the age of 35, between March 2019 and February 2020.[1] Canada also reported 20 cases, of which 40% self-reported vaping THC,[2] and the UK reported two possible cases, both of which were fatal and associated with use of vaping synthetic cannabis.[3] In the US, cases rose sharply in August 2019 and peaked in September 2019. At the time, this prompted many US states and health organisations in the US and globally to warn against the use of e-cigarettes, due to concerns that e-cigarette use was causing the condition.[4] It soon became clear from patient reports and product sample testing that most EVALI cases were linked to a contaminant (vitamin E acetate) present in illicit THC vaping products,[1,5,6] as opposed to legal nicotine e-cigarettes. However, uncertainty about the cause when news of the EVALI outbreak first broke, lack of clarity in terminology,[7] inaccurate reporting in the academic literature,[8] and continued advice by news media[9] and some health authorities[10] to stop use all vaping products rather than THC vapes specifically, even after the cause was identified, appear to have acutely increased perceptions that nicotine e-cigarettes are more harmful than cigarettes.[11–14] There has been comparatively little reporting on the deaths caused by smoking – for example, smoking kills about 75,000 people each year in England.[15] This may have dissuaded people who smoke from switching to e-cigarettes – or even encouraged people already using e-cigarettes to switch (back) to smoking.[11,16,17]

Several studies documented short-term changes in harm perceptions of e-cigarettes following the EVALI outbreak.[11] For example, in England, the nationally-representative Smoking Toolkit Study showed a decline in the proportion of adults who smoke perceiving e-cigarettes as less harmful than cigarettes from before (January-July 2019) to during and immediately after (August-December 2019) the outbreak, while the proportion perceiving e-cigarette use as more harmful rose by more than one-third.[12] Similarly, the International Tobacco Control Policy Evaluation Project (ITC) Youth Tobacco and Vaping Survey[13] also showed a decline in the perception that e-cigarettes are less harmful than cigarettes among 16-19 year-olds across England, Canada, and the US during and immediately after the outbreak, persisting up to August 2020; changes were most pronounced in the US, which had the majority of cases. Likewise, the Health Information National Trends Survey recorded a sharp increase among US adults in perceptions of e-cigarettes as more harmful than cigarettes between January-April 2019 (before the outbreak) and February-June 2020 (after the outbreak).[14]

How harm perceptions of e-cigarettes have continued to change beyond 2020, in the context of the Covid-19 pandemic (since March 2020) and the growing concern about youth vaping (use of disposable e-cigarettes among young people in England has grown rapidly since June 2021[18]) – and the extent to which changes have differed between demographic groups – is not known. We are particularly interested in any differences by age, socioeconomic position, and vaping status. EVALI cases were predominantly young people,[1] and this was reflected in the media coverage at the time.[19] Likewise, concerns about rising prevalence of youth vaping have seen risks to young people emphasised[20] and public health messaging campaigns targeting youth (e.g., the US FDA's 'The Real Cost' campaign)[21]. However, given worries about the impact of vaping on exacerbating Covid-19, a disease affecting the elderly more severely, this may have increased the salience of concerns about vaping in older age group. In addition, evidence suggests older adults are more susceptible to misinformation online.[22,23] As such, it is plausible that trends in harm perceptions over time have

© 2024 Jackson SE et al. *JAMA Network Open.*

differed by age. In terms of socioeconomic position, smoking rates are twice as high among people in low-income occupations compared with those in often higher paid professional and managerial occupations,[24] and consequently tobacco-related morbidity and mortality is more concentrated among disadvantaged groups.[25] People from different socioeconomic groups may also get their information from different news sources.[26] If perceptions differ across the social gradient, targeted corrective health communication messages may help to reduce socioeconomic inequalities in smoking. Finally, it is important to explore differences by vaping status because adults who smoke but do not currently vape are a key target group who could potentially benefit from switching to vaping, while those who vape but continue to smoke may be discouraged from switching completely to vaping if they do not recognise that it is less harmful than smoking.

The Smoking Toolkit Study (a representative monthly survey of adults in England) has been collecting data on harm perceptions of e-cigarettes since before the EVALI outbreak. It is therefore well placed to provide up-to-date descriptive information on perceptions of the relative harms of e-cigarettes compared with cigarettes and insight into trends over recent years.

eReferences

1. Centers for Disease Control and Prevention. Outbreak of Lung Injury Associated with the Use of E-Cigarette, or Vaping, Products. (2020).

2. Government of Canada. Public Health Notice: Outbreak of Vaping-Associated Lung Illness (VALI) from September 2019 to August 2021 in Canada. https://www.canada.ca/en/public-health/services/public-health-notices/2022/outbreak-vaping-associated-lung-illness-september-2019-august-2021.html (2022).

3. Roberts, E., Copeland, C., Robson, D. & McNeill, A. Drug-related deaths associated with vaping product use in the United Kingdom. *Addiction* **116**, 2908–2911 (2021).

4. King, B. A., Jones, C. M., Baldwin, G. T. & Briss, P. A. The EVALI and Youth Vaping Epidemics — Implications for Public Health. *N. Engl. J. Med.* **382**, 689–691 (2020).

5. Blount, B. C. *et al.* Vitamin E Acetate in Bronchoalveolar-Lavage Fluid Associated with EVALI. *N. Engl. J. Med.* **382**, 697–705 (2020).

6. Centers for Disease Control and Prevention. Transcript of December 20, 2019, Telebriefing: Update on Lung Injury Associated with E-cigarette Use, or Vaping. *Centers for Disease Control and Prevention* https://www.cdc.gov/media/releases/2019/t1220_telebriefing_update_lung_injury.html (2019).

7. Cox, S. & Notley, C. Cleaning up the science: the need for an ontology of consensus scientific terms in e-cigarette research. *Addiction* **116**, 997–998 (2021).

8. Shahab, L., Britton, J., Brown, J., Hajek, P. & McNeill, A. The need for an evidence-based and rational debate on e-cigarettes. *The Lancet* **395**, 688 (2020).

© 2024 Jackson SE et al. *JAMA Network Open.*

9. Algiers, O., Wang, Y. & Laestadius, L. Content Analysis of U.S. Newspaper Coverage of Causes and Solutions to Vaping-Associated Lung Injury. *Subst. Use Misuse* **56**, 522–528 (2021).

10. Mendelsohn, C. P., Wodak, A. & Hall, W. Nicotine vaping was not the cause of e-cigarette, or vaping, product use-associated lung injury in the United States. *Drug Alcohol Rev.* **42**, 258–261 (2023).

11. McNeill, A. *et al. Nicotine vaping in England: an evidence update including health risks and perceptions, September 2022. A report commissioned by the Office for Health Improvement and Disparities*. https://www.gov.uk/government/publications/nicotine-vaping-in-england-2022-evidence-update (2022).

12. Tattan-Birch, H., Brown, J., Shahab, L. & Jackson, S. E. Association of the US Outbreak of Vaping-Associated Lung Injury With Perceived Harm of e-Cigarettes Compared With Cigarettes. *JAMA Netw. Open* **3**, e206981 (2020).

13. East, K. *et al.* Exposure to Negative News Stories About Vaping, and Harm Perceptions of Vaping, Among Youth in England, Canada, and the United States Before and After the Outbreak of E-cigarette or Vaping-Associated Lung Injury ('EVALI'). *Nicotine Tob. Res.* **24**, 1386–1395 (2022).

14. Bandi, P. *et al.* Relative Harm Perceptions of E-Cigarettes Versus Cigarettes, U.S. Adults, 2018–2020. *Am. J. Prev. Med.* **63**, 186–194 (2022).

15. NHS Digital. Statistics on Smoking, England 2020. *NHS Digital* https://digital.nhs.uk/data-and-information/publications/statistical/statistics-on-smoking/statistics-on-smoking-england-2020 (2020).

16. Davey, M. 'I've lost my children to vaping': the tragic stories behind the soaring rates of youth addiction. *The Guardian* (2022).

17. Xu, Y., Jiang, L., Prakash, S. & Chen, T. The Impact of Banning Electronic Nicotine Delivery Systems on Combustible Cigarette Sales: Evidence From US State-Level Policies. *Value Health* **25**, 1352–1359 (2022).

18. Tattan-Birch, H., Jackson, S. E., Kock, L., Dockrell, M. & Brown, J. Rapid growth in disposable e-cigarette vaping among young adults in Great Britain from 2021 to 2022: a repeat cross-sectional survey. *Addiction* **118**, 382–386 (2023).

19. Jeong, M. *et al.* Content Analysis of E-cigarette News Articles Amidst the 2019 Vaping-Associated Lung Injury (EVALI) Outbreak in the United States. *Nicotine Tob. Res.* **24**, 799–803 (2022).

20. Mahase, E. Paediatricians call for ban on disposable e-cigarettes as child vaping rises. *BMJ* **381**, p1266 (2023).

21. US Food and Drug Administration (FDA). The Real Cost Campaign. (2023).

22. Allen, J., Howland, B., Mobius, M., Rothschild, D. & Watts, D. J. Evaluating the fake news problem at the scale of the information ecosystem. *Sci. Adv.* **6**, eaay3539 (2020).

© 2024 Jackson SE et al. *JAMA Network Open.*

23. Brashier, N. M. & Schacter, D. L. Aging in an Era of Fake News. *Curr. Dir. Psychol. Sci.* **29**, 316–323 (2020).

24. Office for National Statistics. Adult smoking habits in England: 2021. https://www.ons.gov.uk/peoplepopulationandcommunity/healthandsocialcare/healthandlifeexpectancies/datasets/adultsmokinghabitsinengland (2022).

25. Jha, P. *et al.* Social inequalities in male mortality, and in male mortality from smoking: indirect estimation from national death rates in England and Wales, Poland, and North America. *The Lancet* **368**, 367–370 (2006).

26. Fortunati, L., Deuze, M. & de Luca, F. The New About News: How Print, Online, Free, and Mobile Coconstruct New Audiences in Italy, France, Spain, the Uk, and Germany*. *J. Comput.-Mediat. Commun.* **19**, 121–140 (2014).

© 2024 Jackson SE et al. *JAMA Network Open.*

eTable 1. Harm Perceptions, Aggregated Across Survey Waves, Excluding Don't Know Responses (Unweighted n=24,088)

	% [95% CI]		
	**Less harmful**	**Equally harmful**	**More harmful**
All adults who smoke	41.3 [40.6-42.0]	43.0 [42.3-43.7]	15.7 [15.2-16.2]
Age			
18-34	37.8 [36.8-38.9]	44.4 [43.3-45.5]	17.8 [16.9-18.6]
35-64	44.6 [43.6-45.5]	41.2 [40.2-42.2]	14.2 [13.6-14.9]
≥65	40.6 [38.7-42.5]	45.7 [43.8-47.6]	13.7 [12.4-15.0]
Occupational social grade			
ABC1 (more advantaged)	48.8 [47.8-49.8]	38.8 [37.8-39.8]	12.4 [11.7-13.0]
C2DE (less advantaged)	36.2 [35.3-37.1]	45.8 [44.9-46.8]	17.9 [17.2-18.6]
Vaping status			
Non-vaping	35.2 [34.5-36.0]	46.5 [45.7-47.2]	18.3 [17.7-18.9]
Current vaping	61.9 [60.5-63.3]	31.3 [29.9-32.6]	6.8 [6.1-7.5]

© 2024 Jackson SE et al. *JAMA Network Open.*

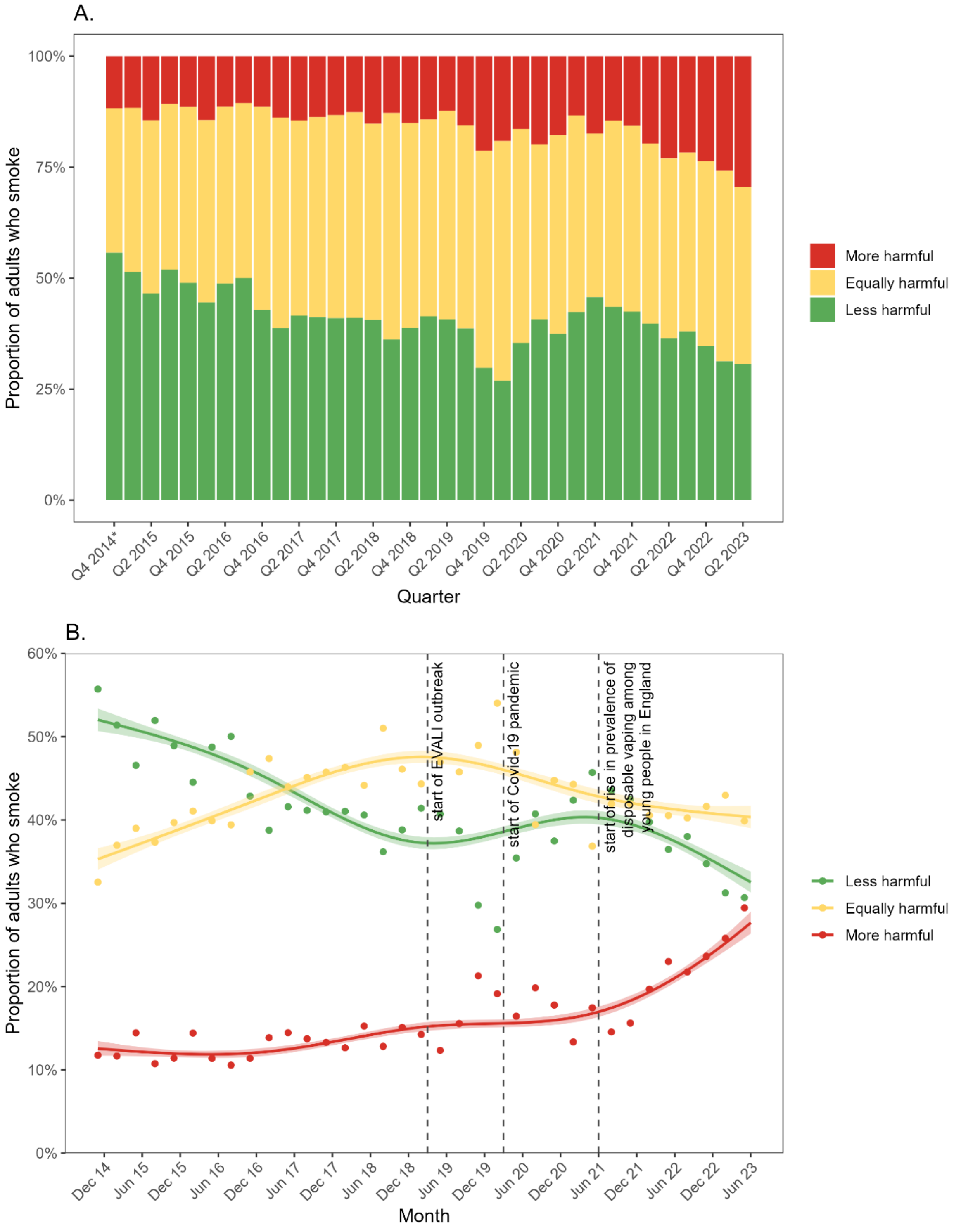

eFigure 1. Harm Perceptions of e-Cigarettes Compared With Cigarettes Among Adults Who Smoke in England, November 2014 to June 2023, Excluding Don't Know Responses (*n*=24,088)

Panel A shows unadjusted weighted proportions, aggregated by quarter. *Q4 2014 includes data from November-December only. Panel B shows modelled monthly time trends: lines represent modelled weighted prevalence by monthly survey wave (modelled non-linearly using restricted cubic splines, five knots); shaded bands represent standard errors; points represent observed weighted prevalence by quarter. Vertical lines indicate the timing of the start of the EVALI outbreak (March 2019), Covid-19 pandemic (March 2020), and the rapid rise in prevalence of disposable vaping among young people in England (June 2021).

© 2024 Jackson SE et al. *JAMA Network Open.*

eTable 2. Changes in Harm Perceptions From the Start to the End of the Study, Excluding Don't Know Responses

	Less harmful			Equally harmful		
	% [95% CI]		Prevalence ratio Nov 14 – Jun 23 [95% CI]	% [95% CI]		Prevalence ratio Nov 14 – Jun 23 [95% CI]
	November 2014[1]	June 2023[1]		November 2014[1]	June 2023[1]	
All adults who smoke	52.0 [49.4-54.7]	32.5 [30.1-35.0]	0.63 [0.57-0.68]	35.3 [32.8-37.8]	40.4 [37.7-43.0]	1.14 [1.04-1.25]
Age						
18-34	45.2 [41.2-49.2]	29.3 [25.6-33.3]	0.65 [0.56-0.76]	38.3 [34.5-42.2]	39.0 [34.9-43.3]	1.02 [0.88-1.17]
35-64	58.4 [54.6-62.1]	34.4 [30.7-38.0]	0.59 [0.52-0.66]	31.2 [27.8-34.7]	40.2 [36.4-44.1]	1.29 [1.13-1.49]
≥65	48.0 [40.0-56.0]	33.5 [26.5-41.4]	0.70 [0.53-0.92]	45.8 [38.0-53.8]	48.7 [40.4-57.0]	1.06 [0.84-1.36]
Occupational social grade						
ABC1 (more advantaged)	62.9 [58.6-67.0]	33.6 [30.4-37.0]	0.53 [0.48-0.60]	28.9 [25.2-33.0]	41.4 [37.9-44.9]	1.43 [1.21-1.68]
C2DE (less advantaged)	46.4 [43.1-49.6]	30.8 [27.2-34.6]	0.66 [0.58-0.76]	38.6 [35.5-41.8]	39.8 [35.9-43.9]	1.03 [0.91-1.18]
Vaping status						
Non-vaping	47.1 [44.1-50.1]	24.0 [21.4-26.8]	0.51 [0.45-0.58]	38.5 [35.7-41.4]	42.1 [38.8-45.4]	1.09 [0.98-1.21]
Current vaping	68.8 [63.3-73.9]	45.7 [40.9-50.6]	0.66 [0.59-0.75]	24.3 [19.8-29.6]	38.6 [33.9-43.5]	1.59 [1.26-2.00]

	More harmful		
	% [95% CI]		Prevalence ratio Nov 14 – Jun 23 [95% CI]
	November 2014[1]	June 2023[1]	
All adults who smoke	12.5 [10.9-14.4]	27.7 [25.2-30.3]	2.20 [1.87-2.59]
Age			
18-34	16.4 [13.7-19.5]	32.7 [28.4-37.2]	1.99 [1.60-2.48]
35-64	10.4 [8.3-12.8]	26.0 [22.5-29.8]	2.51 [1.97-3.23]
≥65	6.4 [3.5-11.5]	17.8 [12.6-24.5]	2.76 [1.46-5.63]
Occupational social grade			
ABC1 (more advantaged)	8.0 [6.1-10.5]	26.0 [22.8-29.4]	3.25 [2.40-4.43]
C2DE (less advantaged)	14.9 [12.8-17.4]	29.7 [26.0-33.8]	1.99 [1.63-2.45]
Vaping status			
Non-vaping	14.3 [12.4-16.4]	34.7 [31.4-38.2]	2.43 [2.07-2.86]
Current vaping	6.7 [4.3-10.4]	16.8 [13.1-21.4]	2.50 [1.55-4.33]

[1] Weighted prevalence from logistic regression models on all adults who smoke and (for estimates by age, occupational social grade, and vaping status) allowing an interaction between survey wave and the moderator of interest, modelled non-linearly using restricted cubic splines.

© 2024 Jackson SE et al. *JAMA Network Open.*

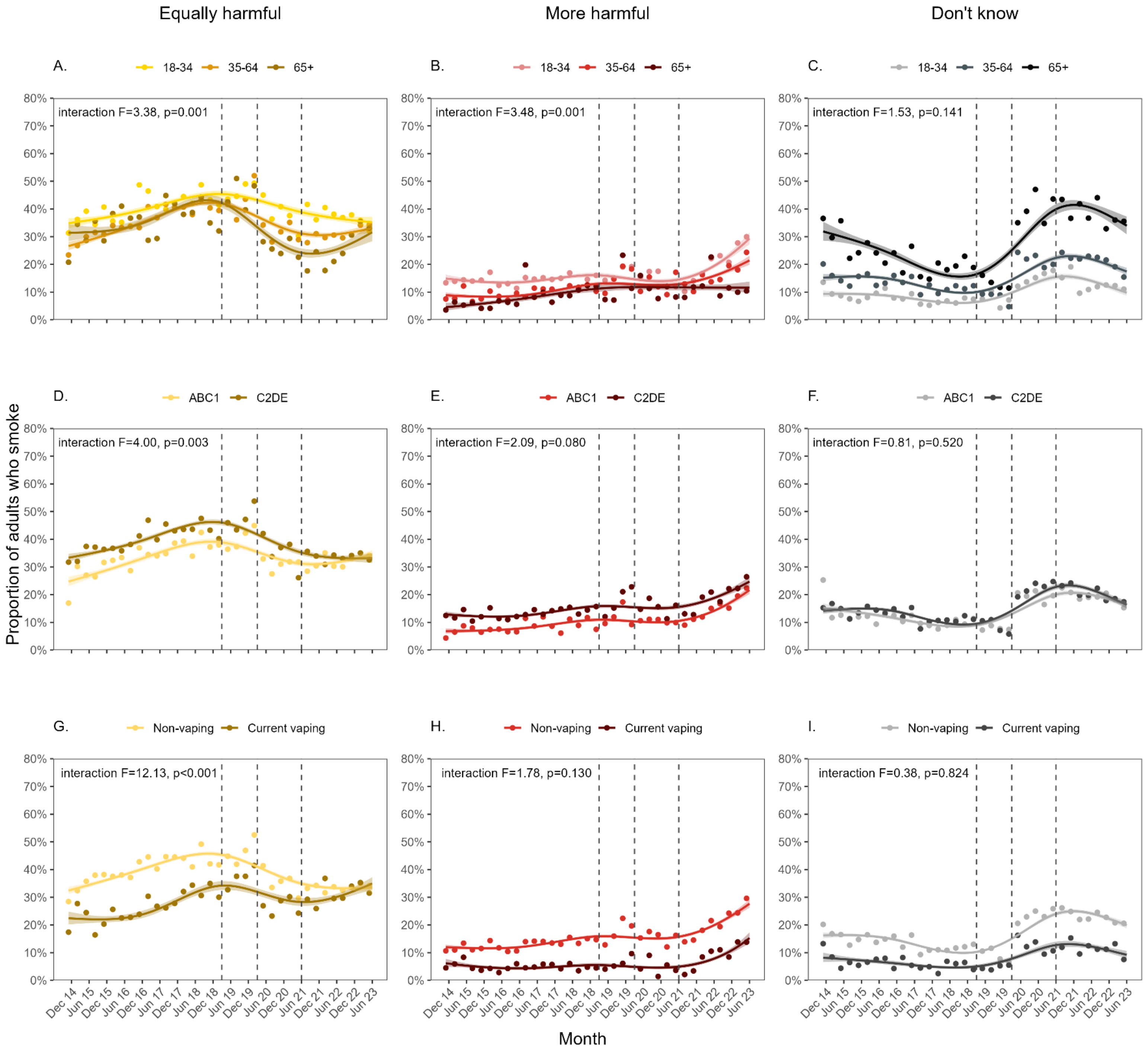

© 2024 Jackson SE et al. *JAMA Network Open.*

eFigure 2. Trends in Harm Perceptions of e-Cigarettes Compared With Cigarettes Among Adults Who Smoke in England, by Age (A-C), Occupational Social Grade (D-F), and Vaping Status (G-I), November 2014 to June 2023: Equally Harmful, More Harmful, and Don't Know

Lines represent point estimates from logistic regression allowing an interaction between age and survey wave, modelled non-linearly using restricted cubic splines (five knots). Shaded bands represent standard errors. Points represent observed weighted prevalence by quarter. Vertical lines indicate the timing of the start of the EVALI outbreak (March 2019), Covid-19 pandemic (March 2020), and the rapid rise in prevalence of disposable vaping among young people in England (June 2021).

© 2024 Jackson SE et al. *JAMA Network Open.*

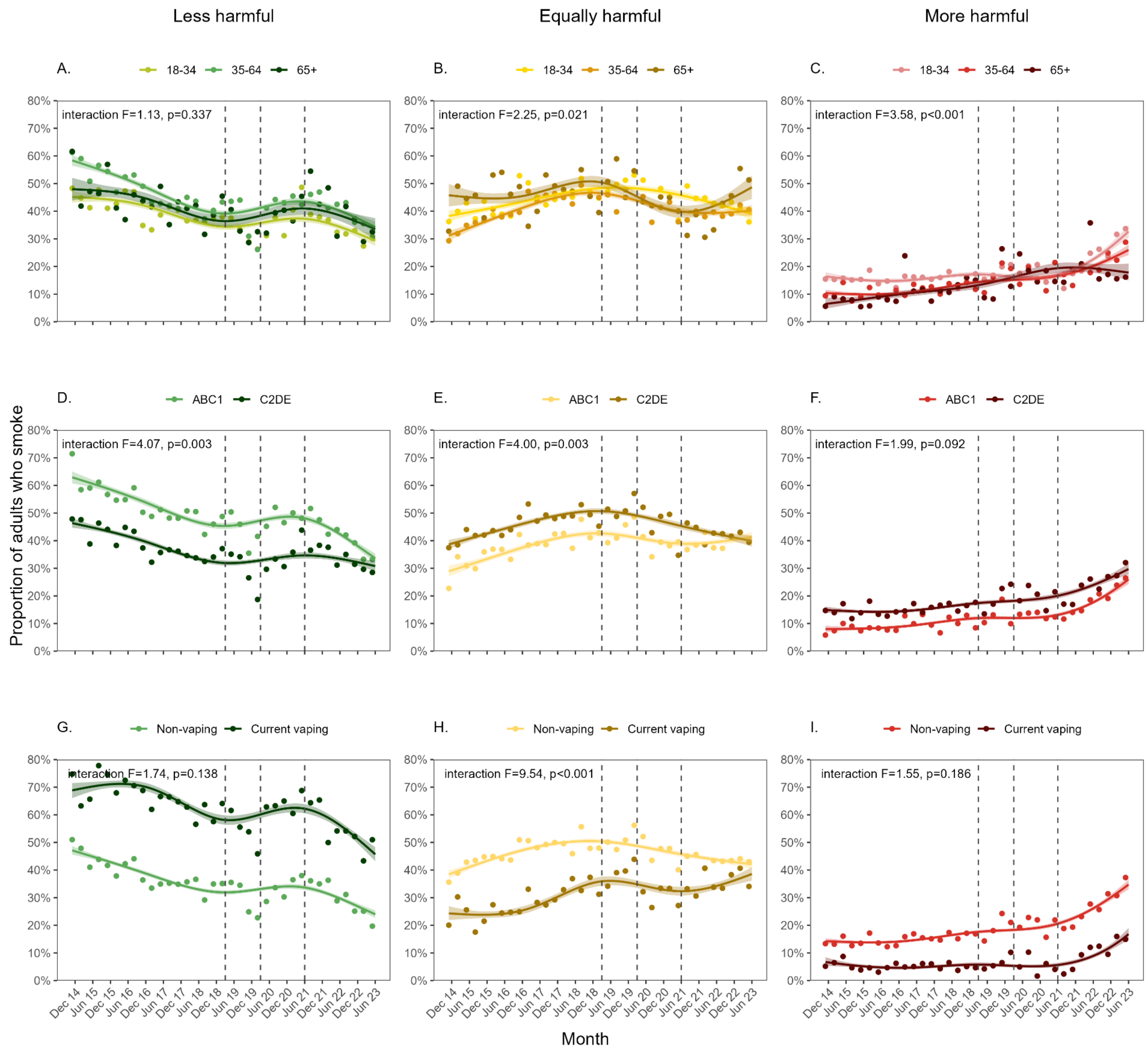
Less harmful
Equally harmful
More harmful
A.
18-34 35-64 65+
interaction F=1.13, p=0.337
B.
18-34 35-64 65+
interaction F=2.25, p=0.021
C.
18-34 35-64 65+
interaction F=3.58, p<0.001
D.
ABC1 C2DE
interaction F=4.07, p=0.003
E.
ABC1 C2DE
interaction F=4.00, p=0.003
F.
ABC1 C2DE
interaction F=1.99, p=0.092
G.
Non-vaping Current vaping
interaction F=1.74, p=0.138
H.
Non-vaping Current vaping
interaction F=9.54, p<0.001
I.
Non-vaping Current vaping
interaction F=1.55, p=0.186
Proportion of adults who smoke
0% 10% 20% 30% 40% 50% 60% 70% 80%
Dec 14 Jun 15 Dec 15 Jun 16 Dec 16 Jun 17 Dec 17 Jun 18 Dec 18 Jun 19 Dec 19 Jun 20 Dec 20 Jun 21 Dec 21 Jun 22 Dec 22 Jun 23
Month

eFigure 3. Trends in Harm Perceptions of e-Cigarettes Compared With Cigarettes Among Adults Who Smoke in England, by Age (A-C), Occupational Social Grade (D-F), and Vaping Status (G-I), November 2014 to June 2023, Excluding Don't Know Responses

Lines represent point estimates from logistic regression allowing an interaction between age and survey wave, modelled non-linearly using restricted cubic splines (five knots). Shaded bands represent standard errors. Points represent observed weighted prevalence by quarter. Vertical lines indicate the timing of the start of the EVALI outbreak (March 2019), Covid-19 pandemic (March 2020), and the rapid rise in prevalence of disposable vaping among young people in England (June 2021).

© 2024 Jackson SE et al. *JAMA Network Open.*
