# Supplementary material for: Trends in Harm Perceptions of E-Cigarettes vs Cigarettes Among Adults Who Smoke in England, 2014-2023
Source: JAMA Netw Open. 2024 Feb 28;7(2):e240582. doi: 10.1001/jamanetworkopen.2024.0582 (PMC10902732; doi:10.1001/jamanetworkopen.2024.0582)
Supplement: Supplement 2. — Data Sharing Statement [file jamanetwopen-e240582-s002.pdf]

Data Sharing Statement

Jackson. Trends in Harm Perceptions of E-Cigarettes vs Cigarettes Among Adults Who Smoke in England, 2014-2023. *JAMA Netw Open*. Published February 28, 2024. doi:10.1001/jamanetworkopen.2024.0582

Data
Data available: Yes
Data types: Deidentified participant data
How to access data: Data are available on Open Science Framework (https://osf.io/wdhru/).
When available: With publication

Supporting Documents
Document types: None

Additional Information
Who can access the data: Anyone can access the data via the link.
Types of analyses: For any purpose.
Mechanisms of data availability: Without investigator support.
Any additional restrictions: The data should not be used for other publications.
